# Health status convergence at the local level: empirical evidence from Austria

**DOI:** 10.1186/1475-9276-10-34

**Published:** 2011-08-24

**Authors:** Martin Gächter, Engelbert Theurl

**Affiliations:** 1 University of Innsbruck, Department of Economics and Statistics; Universitätsstrasse 15, A-6020 Innsbruck, Austria; 2 University of Linz, Department of Economics; Altenberger Strasse 69, A-4040 Linz, Austria; 3 University of Innsbruck, Department of Economics and Statistics; Universitätsstrasse 15, A-6020 Innsbruck, Austria

**Keywords:** mortality, convergence, gender, health status, life expectancy, Austria

## Abstract

**Introduction:**

Health is an important dimension of welfare comparisons across individuals, regions and states. Particularly from a long-term perspective, within-country convergence of the health status has rarely been investigated by applying methods well established in other scientific fields. In the following paper we study the relation between initial levels of the health status and its improvement at the local community level in Austria in the time period 1969-2004.

**Methods:**

We use age standardized mortality rates from 2381 Austrian communities as an indicator for the health status and analyze the convergence/divergence of overall mortality for (i) the whole population, (ii) females, (iii) males and (iv) the gender mortality gap. Convergence/Divergence is studied by applying different concepts of cross-regional inequality (weighted standard deviation, coefficient of variation, Theil-Coefficient of inequality). Various econometric techniques (weighted OLS, Quantile Regression, Kendall's Rank Concordance) are used to test for absolute and conditional beta-convergence in mortality.

**Results:**

Regarding sigma-convergence, we find rather mixed results. While the weighted standard deviation indicates an increase in equality for all four variables, the picture appears less clear when correcting for the decreasing mean in the distribution. However, we find highly significant coefficients for absolute and conditional beta-convergence between the periods. While these results are confirmed by several robustness tests, we also find evidence for the existence of convergence clubs.

**Conclusions:**

The highly significant beta-convergence across communities might be caused by (i) the efforts to harmonize and centralize the health policy at the federal level in Austria since the 1970s, (ii) the diminishing returns of the input factors in the health production function, which might lead to convergence, as the general conditions (e.g. income, education etc.) improve over time, and (iii) the mobility of people across regions, as people tend to move to regions/communities which exhibit more favorable living conditions.

**JEL classification: **I10, I12, I18

## 1 Introduction

It is widely agreed that economic inequality between regions within a country is an important concern from an equity perspective. It is also widely recognized that the focus on regional income as an indicator for economic inequality is too narrow and should be substituted by a broader concept of welfare (see [1,2]). In this respect, empirical studies suggest that (i) health gains have contributed more to human well-being than income growth ([3]) and (ii) the development of the distribution of income differs from the distribution of health. On a world wide scale, the more-than-doubling of life expectancy is probably the most remarkable global human achievement in the last two centuries. This global trend appears to be accelerated in recent decades with life expectancy increasing by more than 10 years between 1963 and 2003. This trend is expected to continue. Until the 1980s, this enhancement was accompanied (i) by a strong convergence in life expectancy within and between countries, (ii) by an increase of the gender gap in favor of females, and (iii) by a substantial transformation of cause-of-death patterns. However, studies based on more recent data indicate that the general improvement in longevity conceals considerable cross-country heterogeneity in many respects (see, for instance, [4]).

Based on the law of diminishing returns, one would expect convergence of the health status due to the existence of upper bounds of many health indicators as well as due to diminishing returns of inputs (e.g. health expenditures, efforts in education, economic development). The reversed effect where countries with higher levels of health experience even faster improvements than other countries is often referred to as the 'Matthew effect' in health (see, for instance, [5,6]). In international studies, convergence of life expectancy is mostly confirmed both for industrialized countries [7] as well as developing countries ([8]). Furthermore, [9-11,4] and [12] show that life expectancy dynamics seem to generate a number of „longevity convergence clubs". Similarly, [13] argue that the data reflect a dynamic pattern that is more complex than a simple convergence process, identifying a bimodal distribution of health from a global perspective. The spread of HIV/AIDS has probably been a significant factor in generating divergence during this period (see [14]). Other studies, however, emphasize that countries with low infant, child or maternal mortality levels subsequently achieve larger decreases in this variable which would confirm the above mentioned Matthew effect (see, for instance, [15,5] among others). [16] study the time series structure of infant mortality rates for 21 countries and reject the Matthew effect hypothesis for all countries except for Australia and the Netherlands.

Particularly from a long term perspective, the majority of research on the convergence of life expectancy/mortality focuses on between-country-convergence on a worldwide or world region scale (for Europe see [17]). [18] conclude their study with the suggestion to expand this line of research to within-country levels.

In this paper we follow their suggestion and study the convergence/divergence of within-state mortality in Austria. Several previous empirical studies focus on regional differences in the health status within countries by applying various theoretical and statistical approaches. In this line of research, a multitude of authors study differences and trends in regional and local (small area) mortality (see, for instance, [19-21], [22] for the UK, or [23] for Andalusia, and [24] for a discussion of the reliable indicators of mortality). Additionally, there is an even broader literature on socioeconomic determinants of regional mortality and life expectancy (e.g. [20,25]). These studies also address the specific challenges of ecological, contextual, and multilevel analysis in regional epidemiology, e.g. the phenomenon of spatial autocorrelation of mortality, neighborhood problems and boundary issues (see, [26-33] or [34]). However, studies testing for health status convergence/divergence between regions using methods well established in other scientific fields (e.g. economic growth theory) are rare. [5] find a negative correlation between initial infant mortality levels and subsequent changes in this variable for the regions of Canada, while the picture is reversed internationally, indicating a strong Matthew effect. [35] confirm the findings for Canada in the 60s and 70s, whereas the trend is reversed in recent decades. Similarly, [36] observes a steady increase in mortality inequality across US counties from 1983-1999, suggesting the presence of a Matthew effect for the US. On the contrary, [37] confirms conditional convergence among (rural) states in India. Recently, [38] study health status convergence between Spanish provinces and regions by applying the concepts of sigma and beta convergence. They draw their attention to the effects of the decentralization process in the Spanish health care system on within-country health inequality.

We use different concepts of sigma convergence as well as absolute and conditional beta convergence to study health status convergence in Austria based on local (small area) information between 1969 - 2004. As an indicator for the health status we use overall standardized mortality rates. We focus on four indicators of mortality, namely (i) mortality of the whole population, (ii) mortality of females, (iii) mortality of males and (iv) the gap in mortality between males and females. Our study enhances the knowledge on developments in regional inequalities of health in several directions. From a long run perspective, Austria experienced a substantial improvement in the health status of the population in the last 40 years, starting from a rather low level compared to countries of similar socioeconomic levels. So far, only very limited systematic evidence is available whether this impressive improvement is accompanied by a convergence or divergence of local mortality. Thus, we add within-country evidence to the already existing between-country evidence, as suggested by [18]. Earlier studies on intra-country convergence mainly focused on large (and thus, more heterogeneous) countries (such as Canada, US, India, Spain) and their results are rather ambiguous. Therefore, our study focuses on a small and homogeneous country and uses the smallest regional unit (local community) to study the convergence of the health status. This is particularly promising as both the between-country as well as within-country comparison of large countries excludes considerable heterogeneity in the dependent and independent variables. In the context of equity considerations our results could also figure as partial/selective answer to the question whether the efforts to guarantee minimum standards of living independent of the individual location were successful. This was a widely agreed principle of regional policy in Austria in the 1970s. Moreover, following a Bismarckian type of health care systems, health care policy in Austria, up to the 1960s, was highly decentralized and particularized. Starting in the 1970s, an important building block of health care reforms in Austria was the harmonization of health policy at the federal level. Finally, by testing for conditional convergence we are able to gain insights into the shape of health production functions at the local level.

The structure of the paper is as follows. Section two presents the methodological framework, indicators and data used in the paper. Section three presents the empirical results including several robustness checks and a discussion of the limitations of the study. Concluding remarks are offered in the final section.

## 2 Methodology and Data

### 2.1 Methodology

To study health status convergence/divergence we use age standardized mortality rates (SMR) for (i) overall mortality of the whole population of a community, (ii) overall mortality of females, (iii) overall mortality of males, and (iv) the gap in overall mortality between males and females. As already mentioned, we apply two widely recognized concepts in economics to study convergence/divergence, namely (i) sigma-convergence and (ii) beta convergence. Three aggregated indicators of between-community inequality are computed to measure sigma-convergence. These measures enable us to track the course of inequality in these health status dimensions and to judge the existence of sigma-convergence or divergence, respectively. The three indicators used are the population-weighted standard deviation (*SD*), the coefficient of variation (*CV*), and a slightly adapted Theil-index of inequality (*L*). Weighted standard deviations are calculated by the root of weighted squared deviations from a community's mortality from the (weighted) mean in the sample. To adjust for the decrease in the mortality mean, the *CV *is calculated from cross section information by dividing a variable's standard deviation *σ *by its mean *μ*, where *σ *and *μ *are averaged over all weighted observation units:

(1) $$CV_{t}=\frac{\sigma_{t}}{\mu_{t}}$$

The Theil-index *L *is defined in the following way:

(2) $$L=\overset{n}{\underset{i=1}{\sum }}p_{i}*ln\;\left(\frac{p_{i}}{x_{i}}\right)$$

where *p_i _*is the population share of local community *i*, ln denotes the natural logarithm, and *x_i _*is the share of the region in the aggregated variable (mortality). *x_i _*is defined as:

(3) $$x_{i}=\frac{P_{i}*y_{i}}{\overset{n}{\underset{i=1}{\sum }}P_{i}*y_{i}}$$

where *P_i _*denotes the population in local community *i *and *y_i _*refers to its mortality. *L *= 0 signals equality, *L >*0 inequality. A decrease (increase) in *L *therefore indicates convergence (divergence).

While the concept of sigma-convergence focuses on the overall spread of the mortality distribution, the concept of (absolute and conditional) beta-convergence relates the change in mortality rates to the starting level, implying an inverse correlation between the starting values and the rates of change. Thus, beta-convergence is a necessary condition for the existence of sigma-convergence, while sigma-convergence might not accompany beta convergence. These concepts were first developed within the framework of neoclassical growth models to explain the convergence in aggregate output between states (regions) (see for example [39]). In these models a common steady state in economic development (absolute convergence) results from the law of diminishing returns of capital inputs. Similarly, health status convergence across regions could be caused by diminishing returns to factor inputs in a regional health production function.

The empirical work on beta-convergence (see [40]) stresses the role of differences in the characteristics of countries (e.g. productivity, quality of education etc.), resulting in the concepts of conditional convergence and convergence clubs (see, for instance, [9]). Both concepts deny common steady states in the economic development. In our context this basically leads to two questions, namely (i) why regions may differ in their health status, and (ii) why such regional differences are expected to decrease (i.e. converge) over time. Regarding the first question, we expect mortality differences between regions due to disparities in terms of the input factors in the regional health production function, such as education, income, household structures, institutional aspects, health care provision, economic development (particularly urban vs. rural areas), and environmental factors. Furthermore, external shocks may lead to such differences, e.g. deviations in immigration rates across regions. With respect to the second question, due to the increasing harmonization and centralization of health policy at the federal level since the 1970s, we would expect convergence of mortality rates (i.e. health status) across communities over time. Moreover, the diminishing returns of the input factors in the health production function might lead to convergence, as the general conditions (e.g. income, education etc.) improve over time. The mobility of people across regions might have a similar effect, as people tend to move to regions/communities which exhibit more favorable living conditions. Putting all these arguments together, contrary to the Matthew effect, we would expect health status convergence across communities (regions) over time.

To measure absolute beta convergence in a cross section of local communities, we employ the following statistical model,

(4) $$ln\left(\frac{{y_{i}}_{,T}}{y_{i,0}}\right)=\alpha +\beta *ln\left(y_{i,0}\right)+\varepsilon_{i}$$

where *y_i, T _*is the mortality (mortality gap) in local community *i *at final time *T*, and *y*_*i*,0 _is the level of mortality in the starting period. *i *corresponds to the local community as the cross sectional unit and *β *pictures the convergence coefficient, where *ε_i _*represents an error term. Equation (4) examines absolute convergence/divergence in the cross section.^1 ^Conditional beta-convergence is estimated by the following equation:

(5) $$ln\left(\frac{y_{i},T}{y_{i,0}}\right)=\alpha +\beta *ln\left(y_{i,0}\right)+\gamma *z_{i,0}+\varepsilon_{i}$$

Thereby *z*_*i*,0 _features characteristics of the local communities (education level, socio-economic level) at time *t *= 0 as further explanatory variables. Thus, they allow the convergence of regions to different steady states due to differences in the input factors of the health production function with respect to the level of education, household structures, economic development, income, or population origins etc. Thus, we assume that differences in the environmental conditions at time *t *= 0 influence the dynamics of convergence across communities.

Health policy, to some extent, is focused (should be focused) on the health status of marginal groups. It is known from previous empirical research that the tails of the mortality distribution might develop in a way that standard regression methods are not able to picture in a proper way, e.g. the existence of convergence clubs separated by different levels of the dependent and independent variable. Accordingly, we also estimate quantile regressions for different segments of the conditional distributions of the change in mortality. The two segments on which this study focuses in this respect are the top (75 percent) and bottom quartiles (25 percent) of the distribution (for technical details of quantile-regression see, for instance, [41]).

### 2.2 Data

As already mentioned, we use standardized mortality rates (SMR) overall and disaggregated by gender as an indicator for the health status to check for convergence or divergence, respectively. Age-standardized mortality rates are available for Austria at the local community level from the *Atlas of Mortality in Austria by Causes of Death *([42]).^2 ^Official death records include the information on the place of residence, age, sex, and cause of death. This information is combined with the results of the population census to calculate the corresponding SMR. To avoid a high dispersion in the SMR (and thus, random variation) caused by small numbers, mortality rates sorted by age and gender are calculated for longer time periods in the official statistics, namely 16 years for the first period (1969-84) and 17 years for the second period (1988-2004). This procedure also excludes year-specific effects (e.g. mortality changes caused by short-term demographic or economic shocks). It also masks possible developments within the two observation periods. The difference in the age structure between regions and between different time periods are accounted for by age-standardization.^3 ^In the case of Vienna, we had to use mortality data at the district level for the period 1978-84 in order to split up Vienna into its 23 districts. Unfortunately, due to our specific focus on the lowest level of aggregation (i.e. local community), mortality data are not available at an annual frequency or for different age cohorts.

Subsequently, we check for sigma-convergence as well as for absolute and conditional beta-convergence using population weighted OLS regression methods to account for the effects of the differing size of local communities.^4^

To test for conditional convergence we control for factors in the health production function which might lead to multiple steady states. More precisely, we include additional variables from the population census 1971 (at *t *= 0) explained below as explanatory variables. As we measure average mortality over two longer time periods, the smoothing of year-specific effects should be accounted for in the selection of explanatory variables. Thus, we focus on variables with low fluctuations over time. More precisely, we test for the level of education, the household structure and social attachments, the population origin, the economic development, and the distribution of genetic characteristics^5^:

*• **Level of education: ***To control for the impact of education on mortality, we consider five groups of educational levels. To calculate the average education level in the local community, we multiplied the numbers of persons in each group with the corresponding level of education, and divided the sum of the subgroups by the population above 15 years, as indicated in equation (6),

(6) $$Edu=\frac{\sum_{E=1}^{5}POP_{E}*E}{POP_{15}}$$

where *E *corresponds to the level of education, *POP_E _*is the population in each subgroup, and *POP*_15 _is the overall population above 15 years. The factors used for the educational level were (1) compulsory school, (2) apprenticeship or secondary education, (3) higher school certificate (general qualification for university entrance), (4) an additional education after this school-leaving certificate (e.g. a polytechnic education or a college) excluding university education, and finally (5) a university degree or equivalent.^6 ^Thus, we obtain an index measuring the average educational level, (theoretically) ranging from 1 to 5 within regions where increasing values indicate a higher level of education, respectively. Subsequently, the same procedure was applied to gender-specific educational levels.

*• **Household structure and social attachments: ***There exists a broad literature on the effect of different familial networks on mortality (for an overview see [43]). To control for these effects at the local community level, we proceeded in the following way. We selected the following family related variables from the census 1971, namely

- the average number of people living in a household,

- the share of one-person households,

- the share of households comprising a couple with children,

- the share of households comprising a couple without children, where the woman is 40 or older,

- the share of single-households with children,

- the average number of children per family,

- the share of divorced women, in percent of the ever married, and

- the share of female singles, age 40-59.

As expected, we observe a high correlation between those dimensions. Thus, a principal component analysis seems to be appropriate to convert the various characteristics into one single variable. As we included eight variables in our analysis, and the eigenvalue of the first factor amounts to 5.45, the resulting factor explains approximately 68% of the total variance. Average household size, couples with children, and the average number of children per family are negatively correlated with the factor, while the remaining variables mentioned above influence the factor in the reverse direction (one-person households, couples without children, singles with children, the share of divorced women and the share of female singles in the age between 40 and 59). To sum up, traditional family structures including a couple with children or more people living in a household exercise a negative influence on the factor. On the contrary, one-person households, couples without children, singles with children, and a higher share of divorced or single women increase the resulting factor. By reversing the factor (multiplying it by -1) we are able to interpret the resulting variable as „Social and familial attachments", with increasing values of the factor indicating a higher level of social attachments and familial solidarity, respectively (see [43] for a similar approach).

*• **Population origins: ***Although it was rarely considered in earlier studies on mortality or life expectancy convergence, we include the share of foreigners as an explanatory variable. Previous research shows that mortality is significantly lower in regions with a higher share of immigrants or foreigners (see, for instance, [43,44] or [45]). Common explanations (see [43]) range from selection effects (immigrants might be healthier) to the meaning of voluntary migration (taking control of one's life) and to the solidarity created within marginalized migrant communities. Thus, it also seems appropriate to include this variable in a convergence equation as a conditional variable.

*• **Economic Development: ***As data on average income are not available at the local community level for the starting period, we use two proxies to measure the economic development in a community: labor force participation rates and commuters. Depending on the estimation, we use the overall or gender-specific participation rate as explanatory variable. In the case of commuters, we calculated the ratio of in-commuters (who live outside and commute into the community) and the community population. The higher this ratio, the higher the economic level, as more jobs are available in those communities.

*• **Genetic structure of the population: ***The genetic characteristics of individuals are one important input in the health status production function from an individual point of view. If aggregates of individuals (e.g. regions) are compared, the genetic structure is either seen as homogeneous or controlled for by proxies such as ethnic criteria or the population origin. We control for the genetic structure of the local communities in the following way. [46] offer data on the genetic structure of the Austrian population based on a surname analysis (see also [47] for an application of this method to explain differences in suicide rates in Austria on the district level). For societies with patrilinear surnames the surname can be considered as a single gene with a multitude of neutral alleles which are transmitted as in unisexual haploid species ([46]). Surnames can be considered as close substitute for Y-chromosome markers and haplotypes. [46] used information of about 4 million telephone users to calculate the surname frequency distribution for the 120 largest Austrian towns. Statistical classification of surname occurrence and frequency patterns yielded five major regions reflecting the genetic structure of the population. We assigned the Austrian communities to these five genetic regions on the level of districts. Region I includes the southern parts of the province of Salzburg, the eastern parts of Tyrol, the southern parts of Lower Austria, Styria and the northwestern parts of Carinthia. Region II includes Upper Austria and the northern parts of Salzburg. Region III includes the north and eastern parts of Lower Austria, Vienna and Burgenland. Region IV includes the central and western parts of Tyrol and Vorarlberg. Region V includes the central and eastern parts of Carinthia. Within the regression this information is used as dummy information, whereas region III (the north and east of Lower Austria, Vienna and Burgenland) is the reference region.

The following section presents important descriptive statistics and the empirical results of our examination of health status convergence across regions.

## 3 Main Results

### 3.1 Descriptive Statistics

Summary statistics both of our dependent as well as explanatory variables for the first period (1969-84) and the population census in 1971 are reported in Table 1. Means and standard deviations are weighted by community size. Male mortality is considerably higher (1392.273) than female mortality (855.070). On the contrary, the growth rates of male and female mortality differ only slightly. However, as the growth rates are measured in percentage points, male mortality is nevertheless decreasing more quickly in absolute terms, which can also be seen by means of the negative growth rate for the gender mortality gap (-5.282%). The average share of foreigners amounts to 2.8%, the labor participation rates exhibits a value of 41.74%. The in-commuter ratio is quite high, amounting to 9.85%.

**Table 1:** Summary statistics (community level)

Variable	Mean	**Std. Dev**.	**Min**.	**Max**.
SMR, overall	1069.264	159.124	533.007	2693.433
SMR, growth rate	-32.594	8.321	-74.739	82.573
Male SMR	1392.273	192.674	501.927	2875.495
Male SMR, growth rate	-32.276	8.697	-76.765	189.509
Female SMR	855.070	157.059	402.022	2508.721
Female SMR, growth rate	-33.335	10.619	-92.913	80.362
Gender Mortality Gap	537.203	146.849	-1023.080	1814.17
Gender Mortality Gap, growth rate*	-5.828	1069.361	-98.530	84084.672
Education, average	1.509	0.246	1.020	2.290
Social attachments	0.494	0.901	-1.217	3.415
Foreigners, share	2.828	2.566	0.000	33.202
Labor participation rate	41.740	3.280	26.700	62.300
Commuter ratio	9.850	9.910	0.000	138.001

*Notes: *Means and standard deviations are weighted by population. All reported values correspond to the first period (1969-1984), while the socioeconomic variables are taken from the census 1971. Growth rates report the percentage change from period one (1969-84) to period two (1988-2004).

*In the case of this variable, we excluded observations with negative values in one of the two periods. Thus, the reported values include only 2324 (out of 2381) communities.

**Figure 1:**
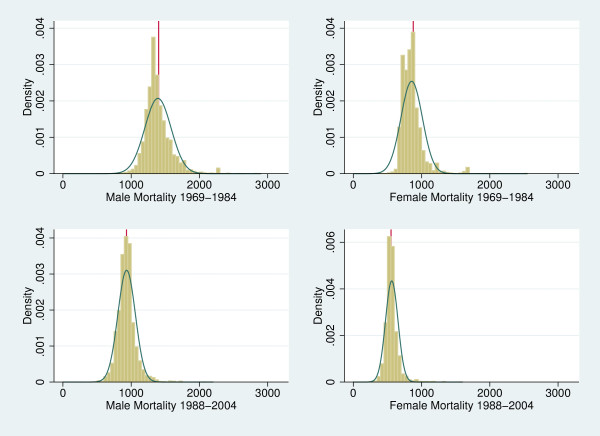
**Mortality levels in period one (1969-1984) and two (1988-2004)**.

Figure 1 shows the development of female and male mortality levels over time. We observe a substantial improvement in the health status both among males and females, while it seems unclear whether the variation of mortality levels increases or decreases.

**Figure 2:**
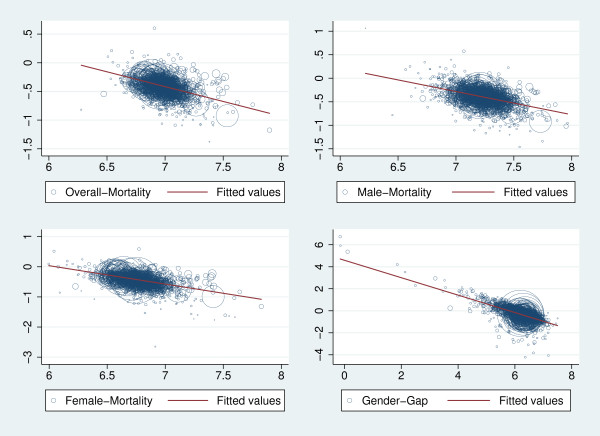
**Scatter plots for the level of each variable in period one (abscissa in logs) and the corresponding percentage change (ordinate in log-differences) from period one to two**. The magnitude of the circle shows the size of the community (population).

Figure 2 shows scatter plots of the mortality levels and the corresponding growth rate of the variable from period one to two. At first sight, each of the four figures shows a negative relationship (as shown by reference to the regression line), which indicates absolute convergence of mortality from period one to two.

### 3.2 Empirical Results: Sigma- and Beta-Convergence

We start with the examination of the patterns of cross-regional dispersion or inequality by calculating the weighted standard deviations, the coefficient of variation, and the Theil-index of inequality for each variable (overall mortality, mortality by gender and the resulting gender gap) and each period. The values of these indicators are reported in Table 2. For each variable, the first column reports the weighted standard deviation (*σ*), the second column the coefficient of variation (CV) and finally, the Theil-index of inequality (L). For all four variables, the weighted standard deviation shows an increase in equality, as the value decreases from period one to two. However, the picture seems less clear when correcting for the (falling) mean in the distribution. Both the coefficient of variation and the Theil-index of inequality (L) do hardly change, and in some cases (male mortality, overall mortality) even increase from period one to two. Despite of these mixed results for sigma-convergence, it is also important to analyze absolute and conditional beta-convergence, as it is based on a different methodological framework of examining convergence.

**Table 2:** Empirical Results - *σ*-Convergence

Method	*σ*		CV		L	
**Period**	**1**	**2**	**1**	**2**	**1**	**2**

**SMR Males**	192.6739	128.5883	0.1384	0.1377	0.0087	0.0103
**SMR Females**	157.0589	91.9134	0.1837	0.1631	0.0139	0.0134
**SMR Overall**	159.1242	98.1804	0.1488	0.1371	0.0096	0.0099
**Gender Gap**	146.8487	97.1865	0.2734	0.2626	--	--

*Notes: σ *reports weighted standard deviations, whereas *CV *corresponds to the *Coefficient of variation *and *L *reports the *Theil-index of inequality*, respectively. In the case of *CV*, weighted standard deviations were used. *L *could not be calculated in the case of the gender mortality gap due to negative values of this variable in 57 cases, where the natural logarithm could not be calculated.

Weighted OLS estimation results for *β*-convergence are shown in Table 3, where the first column for each variable reports absolute beta-convergence, as specified in equation (4), and the second column shows the results for conditional beta-convergence, as constructed in equation (5).

**Table 3:** Empirical Results - Absolute and Relative *β*-Convergence

Dependent variable	Change SMR		Change SMR Males		Change SMR Females		Change Gender Gap	
Method	Absolute *β*	Relative *β*	Absolute *β*	Relative *β*	Absolute *β*	Relative *β*	Absolute *β*	Relative *β*
*β*-Coefficient	-0.517*** (0.016)	-0.552*** (0.017)	-0.493*** (0.018)	-0.617*** (0.019)	-0.610*** (0.017)	-0.589*** (0.017)	-0.792*** (0.018)	-0.868*** (0.018)
Education, average		-0.242*** (0.018)		-0.259*** (0.016)		-0.220*** (0.027)		-0.707*** (0.049)
Social attachments		-0.065*** (0.005)		-0.067*** (0.006)		-0.061*** (0.007)		-0.172*** (0.014)
Foreigners, share		-0.004*** (0.001)		-0.006*** (0.001)		-0.004*** (0.001)		-0.012*** (0.003)
Participation rate, share		-0.002*** (0.001)		-0.008*** (0.001)		-0.001 (0.001)		-0.009*** (0.002)
Commuters		0.002*** (0.000)		0.002*** (0.000)		0.001*** (0.000)		0.004*** (0.001)
Genetic 1		-0.035*** (0.006)		-0.020*** (0.007)		-0.046*** (0.008)		0.020 (0.017)
Genetic 2		-0.030*** (0.006)		-0.016** (0.007)		-0.039*** (0.008)		0.035** (0.017)
Genetic 3		-0.033*** (0.010)		0.004 (0.010)		-0.059*** (0.013)		0.111*** (0.026)
Genetic 4		-0.037*** (0.010)		-0.021* (0.011)		-0.047*** (0.013)		0.012 (0.027)
Constant	3.204*** (0.114)	3.962*** (0.131)	3.169*** (0.130)	4.970*** (0.178)	3.696*** (0.115)	3.917*** (0.123)	4.588*** (0.110)	6.575*** (0.182)

N	2381	2381	2381	2381	2381	2381	2324	2324
*R*^2^	0.297	0.395	0.239	0.347	0.350	0.423	0.467	0.530

*Notes: *The first value reports regression Coefficients, standard errors are reported in parentheses. *, **, *** denote 10%, 5% and 1% significance levels. Regressions are weighted by community size (population). For the variables *„Education, average" *and *„Participation rate, share" *gender-specific values were used for gender-specific mortality convergence. The four categories for genetic structures are dummy variables following the study by [46], where the region III (North and East of Lower Austria, Vienna, Burgenland) is the reference region.

For all observed variables, we find highly significant coefficients for absolute and conditional beta-convergence from period one to two. Interestingly, while the male coefficient is smaller than the female in the absolute convergence specification, it exceeds the female coefficient when controlling for other factors. Similarly, in communities which exhibit a high gender mortality gap in the first period, the decrease in this variable is much higher as compared to regions where the gender mortality gap was already smaller in the first period.^7 ^As expected, a higher educational level, stronger social attachments, and a higher share of foreigners accelerate the decrease in mortality both for males and females. The same applies to the labor participation rate, although it appears insignificant in the estimation for female mortality. The commuter ratio (and thus, a higher economic development) exercises a positive influence on the growth rate, and thus, seems to slow down the improvement in terms of mortality and life expectancy, respectively.

The results for our dummy variables for the genetic structure seem to be particularly interesting. For the change in the SMR for the whole population all genetic regions show a significant negative coefficient of similar size compared to the reference region (Northern and Eastern Lower Austria, Vienna, Burgenland). As the region including Vienna served as base category, our results could be reversed by taking a different region as base category. Thus, as the magnitude of the coefficients across regions feature similar values in Table 3, just the region of Vienna (and the surroundings as explained above) show significant coefficients in this slightly changed specification (not shown).

### 3.3 Robustness Checks and Discussion

To substantiate our empirical results we proceed in the following way. (i) We run robustness tests in several directions and discuss their results below. (ii) We refer to potential limits of our study and discuss strategies for improvement. We start with the robustness checks.

Regarding the significant influence of the genetic structure, we tried a specification where we included eight dummy variables for the nine federal states (*Bundesländer*) in Austria. While we concluded from our regressions above that the remaining regions (other than Vienna, Burgenland and Lower Austria) experience a higher decrease in mortality from period one to two due to the specific genetic structure, this effect could also be due to other unobserved regional characteristics. Not surprisingly, the effects appear mostly insignificant when including eight dummy variables for the nine federal states in Austria in our conditional beta-convergence estimations (not shown). However, this is not surprising taking into account that we distinguished five different genetic regions (including four dummies), while the state effects include eight regional dummy variables. Thus, although it is not appropriate to conclude that the genetic structure plays a major role in our analysis, it is nevertheless one plausible explanation for the observed regional effects.

To test for the hypothesis of the well-recognized pattern of „convergence clubs" (as described above), we also ran separate regressions for each of the five genetic regions. Interestingly, when testing for absolute beta-convergence, there is a considerable difference between the coefficients (not shown). More precisely, the growth rate of mortality depends more strongly on the initial value in region III, which was our baseline (omitted) category in the estimations above. Thus, while it seems that mortality decreases at a lower pace in this region (as the dummies for other regions show significantly negative coefficients), we also find a stronger relationship between the initial mortality rate and subsequent growth rates. This applies to overall mortality as well as mortality by gender, while the differences between regions in terms of the gender mortality gap are less strongly pronounced. Moreover, the differences between other regions are also of considerable magnitude, albeit less distinctive. Clearly, this result backs the above mentioned convergence clubs hypothesis. However, a detailed analysis of the differences between those regions would go beyond the scope of this paper. Despite of these differences, the robustness of our results presented in the preceding section is confirmed, as we find a significant beta-convergence in each region and dependent variable.

The well-known „regression to the mean" problem might cause considerable bias in regressions of absolute and conditional beta-convergence as presented above. Thus, the methodology of 'Barro regressions' (as applied in the last section) was criticized by [48] and [49], who emphasize that this method is subject to Galton's fallacy. We controlled for this problem in several ways. (i) We applied weighted regressions according to the population size of the communities (as explained above). (ii) We observed long term mortality and eliminate short time effects, which might be strongly influenced by the stochastic component. (iii) Finally, we also ran regressions excluding smaller communities with less than 500 inhabitants to account for this random variation (not shown). Once again, the results only changed slightly, confirming significant beta-convergence between communities in Austria.

[50] propose an alternative strategy to test for beta-convergence, which is sometimes also referred to as gamma-convergence. They argue that sigma- and beta-convergence measure two different dimensions of convergence. While the former measures convergence by simply tracking the intertemporal change in the coefficient of variation, the latter is concerned with the intra-distributional mobility over time. More precisely, we examine the change in the ranking of communities with respect to mortality. The measure focuses on the evolution of the ordinal ranking over a particular time interval. In their paper, they suggest a slightly adapted Kendall's index of rank accordance (see [51]) to measure beta-convergence. In a binary version, we focus on the concordance between the ranks in year *t *and year 0. This rank concordance index is calculated by:

(7) $$RC=\frac{Variance\left(AR{\left(Y\right)}_{it}+AR{\left(Y\right)}_{i0}\right)}{Variance\left(2*AR{\left(Y\right)}_{i0}\right)}$$

where *AR*(*Y*)*_it _*is the actual rank of community *i*'s mortality rate in year *t *(period 2), and *AR(Y)*_*i*0 _the rank of community *i*'s mortality rate in year 0 (period one). The value of this index ranges from zero to unity, where the denominator is the maximum sum of ranks which would be obtained if there were no change in rankings over time. The closer the index is to zero the greater the extent of mobility within the distribution ([50], p. 259]). The values for this *rank concordance index *are reported in Table 4.

**Table 4:** Empirical Results - Rank Concordance Index

Variable	SMR Males	SMR Females	SMR Overall	Gender Gap
RC	0.7336	0.5916	0.6450	0.6989

*Notes: *The *Rank Concordance Index *(following [50]) was calculated by using population-Weighted variances as described in equation (7).

The rank concordance measure also confirms beta-convergence in mortality rates between communities in Austria, while the convergence seems to be stronger among females as compared to males.^8 ^As noted by [50], the simple measure does not tell anything about the dynamics of evolving mortality distributions. An in-depth analysis of the changes in the distribution, however, would be particularly interesting in our context, because it could reveal helpful information to test for the persistence of relative mortality advantages/disadvantages. Thus, Table 5 shows the rank changes in overall mortality from period one to two.

**Table 5:** Empirical Results - Rank Changes (Overall Mortality)

Percentile	10	20	30	40	50	60	70	80	90	100	Total
10	79 33.19	45 18.91	20 8.40	22 9.24	13 5.46	22 9.24	7 2.94	8 3.36	9 3.78	13 5.44	238 10.00

20	43 18.07	39 16.39	30 12.61	29 12.18	26 10.92	11 4.62	14 5.88	16 6.72	14 5.88	16 6.69	238 10.00

30	31 13.03	43 18.07	31 13.03	30 12.61	21 8.82	19 7.98	21 8.82	17 7.14	16 6.72	9 3.77	238 10.00

40	33 13.87	27 11.34	30 12.61	25 10.50	23 9.66	27 11.34	18 7.56	22 9.24	27 11.34	6 2.51	238 10.00

50	16 6.72	21 8.82	31 13.03	34 14.29	30 12.61	30 12.61	20 8.40	19 7.98	22 9.24	15 6.28	238 10.00

60	11 4.62	20 8.40	26 10.92	31 13.03	32 13.45	24 10.08	32 13.45	21 8.82	25 10.50	16 6.69	238 10.00

70	9 3.78	14 5.88	19 7.98	27 11.34	29 12.18	28 11.76	42 17.65	29 12.18	23 9.66	18 7.53	238 10.00

80	4 1.68	13 5.46	20 8.40	16 6.72	24 10.08	36 15.13	30 12.61	45 18.91	25 10.50	25 10.46	238 10.00

90	7 2.94	8 3.36	22 9.24	17 7.14	24 10.08	24 10.08	36 15.13	28 11.76	32 13.45	40 16.74	238 10.00

100	5 2.10	8 3.36	9 3.78	7 2.94	16 6.72	17 7.14	18 7.56	33 13.87	45 18.91	81 33.89	239 10.04

Total	238 100.00	238 100.00	238 100.00	238 100.00	238 100.00	238 100.00	238 100.00	238 100.00	238 100.00	239 100.00	2,381 100.00

*Notes: *The mortality deciles of the first period (1969-1984) are reported in rows, where the deciles of the second period (1988-2004) are reported in columns. The first value reports absolute values, the second percentages (on the basis of 238 communities per decile).

For calculating this cross table, we divided our sample in period one into ten deciles of 238 communities each (and 239 in the last decile, as we have a total of 2381 communities). The first decile (as reported as „10") refers to the decile with the highest mortality in period one. The columns show the deciles of mortality rates in period two. Thus, the table shows the change in rankings depending on the original decile of the community, giving further insights into rank changes besides the aggregated measure of the Rank Concordance Index. The results indicate, for instance, that 33.19% (or 79 out of 238 communities) which have been in the first decile in period one also remained there in period two. On the contrary, 13 communities (5.44%) changed from the first to the last decile (and thus, from the highest mortality decile to the lowest level of mortality). In the case of no change in rankings, the diagonal would show values of 100% each, as no community would change the decile from period one to two. In a nutshell, it is easy to see that there were major rank changes from period one to two, leading to our highly significant *rank concordance *measure presented above.

Another common criticism of the Barro-approach is that the growth regressions assume an implicit condition of homogeneity, or in other words, that all communities/regions are restricted to have the same rate of convergence represented by the beta-coefficient. Thus, the process of formation of convergence clubs cannot be captured by using simple Barro regressions, as we only estimate a single *β *for all communities in the sample. This problem as well as the criticism related to Galton's fallacy can partly be handled by applying quantile regressions, because they allow for heterogeneity in the coefficients of the regression. Thus, the phenomenon of „convergence clubs in mortality" could also be revealed by such a methodology. Results for quantile regressions for absolute beta-convergence are reported in Table 6.

**Table 6:** Empirical Results - Quantile Regressions for Absolute *β*-Convergence

**Dep. Var**.	SMR Overall		SMR Males		SMR Females		Gender Gap	
Quantile	0.25	0.75	0.25	0.75	0.25	0.75	0.25	0.75
*β*-Coefficient	-0.668*** (0.076)	-0.456*** (0.049)	-0.539*** (0.051)	-0.334*** (0.045)	-0.689*** (0.044)	-0.555*** (0.029)	-0.660*** (0.037)	-0.832*** (0.019)
Constant	4.194*** (0.531)	2.840*** (0.343)	3.442*** (0.369)	2.078*** (0.328)	4.153*** (0.295)	3.397*** (0.201)	3.644*** (0.229)	4.966*** (0.114)

N	2381	2381	2381	2381	2381	2381	2324	2324

*Notes: *The first value reports regression Coefficients, standard errors are reported in parentheses. *, **, *** denote 10%, 5% and 1% significance levels. Regressions are weighted by community size (population).

In essence, we estimate quantile regressions for different segments of the conditional distribution of the relative decrease in mortality, similarly to the analysis by [4]. As shown in Table 6, the beta-coefficient is higher in the lower quartile (0.25) as compared to the upper quartile (0.75). This pattern is not only observed for overall mortality, but also for gender-specific mortality. Thus, the relationship between the initial level and the growth rate in mortality is stronger in the lower quartile of the conditional distribution, which would once again confirm the existence of convergence clubs. This result does not change when including other explanatory variables by estimating quantile regressions of conditional beta-convergence (not shown, available on request by the authors). Despite this evidence of convergence clubs, we nevertheless are able to conclude that we observe absolute as well as conditional beta-convergence in all our quantile regressions, albeit the speed of convergence differs significantly between different quantiles in the distribution.

Overall, our checks allow the conclusion that the results are quite robust and the methodology used is appropriate for our research question. This leads to a discussion of possible limitations of our study. Our indicator for health status is overall mortality of the total population measured by age standardized mortality rates. No information on life expectancy and quality related aspects of health is currently available at the level of local communities in Austria. For specific health policy conclusions, overall mortality of the total population might mask structural information of two kinds: (i) the mortality caused by different disease groups, and (ii) the mortality ratios of different age groups. Data on the first issue are available and it is up to future research to study the convergence/divergence of disease specific mortality rates. In their study of long term inequalities between British regions, [19] point out that the use of standardized mortality rates might obscure differences in the convergence rates of age specific death rates between regions. A look at mortality rates divided by age groups might also improve the insights into the health production function which forms the basis for conditional convergence. Unfortunately, data on this issue are not available on the local level in Austria. Finally, our study relies on units of observation and a level of data aggregation defined by general administrative boundaries. We are aware that this causes problems in several respects (see [52,27]). The literature on boundary issues (see e.g. [31,34,33,32]) agrees that administrative boundaries are not able to cope with the problem of neighborhood in an appropriate way and favors a multi-perspective approach for defining neighborhood units. On the other hand, previous attempts to implement such approaches in empirical studies (see [31]) allows the conclusion that this strategy is only possible for small scaled projects and not for country wide comparisons.

## 4 Conclusions

Particularly from a long-term perspective, within-country convergence of mortality has rarely been investigated by applying methods well established in other scientific fields, especially in the economic growth literature. In this paper we study within-country convergence of mortality in Austria, a rather homogeneous country. We used data from 2381 Austrian communities from 1969 - 2004 to test for various forms of beta- and sigma-convergence. As an indicator for the health status we used overall standardized mortality rates by considering four different dimensions, namely (i) the overall population, (ii) males, (iii) females, and (iv) the resulting gender mortality gap.

Regarding sigma-convergence, we find rather mixed results. While the weighted standard deviation indicates an increase in equality for all four variables, the picture appears less clear when correcting for the decreasing mean in the distribution (coefficient of variation and Theil-index of inequality). On the contrary, we find highly significant coefficients for absolute and conditional beta-convergence between the periods. The highly significant beta-convergence across communities might be caused by (i) the efforts to harmonize and centralize the health policy at the federal level in Austria since the 1970s, (ii) the diminishing returns of the input factors in the health production function, which might lead to convergence, as the general conditions (e.g. income, education etc.) improve over time, and (iii) the mobility of people across regions, as people tend to move to regions/communities which exhibit more favorable living conditions. While these results are confirmed by several robustness tests, we also find evidence for the existence of convergence clubs. Both the significance of the dummy variables for genetic structures in our conditional beta-convergence estimation as well as the considerable difference of the beta-coefficients when running separate regressions for these regions can be interpreted as evidence for possible convergence clubs. In order to test for differences in the beta-coefficients within the distribution we also ran quantile regressions for the lower and upper quartile of the distribution. Once again, the impression of divergences in the coefficients in different parts of the distribution was confirmed, albeit the conclusion of beta-convergence across communities is unaffected by this result. Our results basically confirm the findings from [5] for Canada and [37] for India, while the studies by [35] and [36] find a significant Matthew effect for Canada and the US in recent decades.

While we use data from Austria, a small and homogeneous country, it would also be interesting to extend this line of research to other countries to test for conditional beta-convergence by including further explanatory variables. Particularly, such studies could give additional insights into the dynamics of mortality developments in large countries (i.e. Canada, US) where previous studies find divergence of health statuses and the presence of a Matthew effect. Finally, it would also be interesting to test other measures of health status in similar regressions. Given the huge contribution of gains in health to overall human well-being in the last decades, such studies are also highly rewarding from a welfare and equity perspective.

## 5 Competing interests

The authors declare that they have no competing interests.

## 6 Authors' contributions

The authors contributed equally to this work. Both authors read and approved the final manuscript.

## Notes

^1^Note that the dependent variable refers to the growth rate from period 0 to period T. The speed of beta-convergence can be calculated from the regression coefficient *β *on the initial level *y*_0_. For the specification at hand, the speed of convergence equals -*ln*(1 + *β*)*/T*.

^2^Following the NUTS-classification, the local community level is LAU2. Vienna is counted as 23 local communities mirroring the districts of Vienna. In the Austrian political system local communities act as agents in the administration of public functions of the central state and the states (e.g. several public health tasks) and fulfill several tasks self-governed. The mean size (population) of the communities in period two (population census 2001) is 3373, the median is 1575. The number and size of communities is based on historical contingencies and not necessarily the result of an optimal spatial organization of public policy (e.g. in the health care sector).

^3^For mortality data at the community level, the method of indirect standardization was used. Although we are aware of the limitations of indirectly standardized mortality rates (see, for instance, [24]), we use them for our analysis as we are able to assume that the age structure across communities is rather homogeneous in Austria, which minimizes possible biases of the method of indirect standardization of mortality rates. Furthermore, we weight our results by community size to account for random variation. For details about the standardization method see [42].

^4^All our regression results are weighted by the community size (population) to account for random variation in our sample. Other weighting procedures have been proposed in the literature, such as an „intermediate" solution between unweighted and fully weighted regressions, as suggested by [53]. More precisely, they take account of three sources of variation in death rates, namely sampling error, explanatory variables and unexplained differences between areas. However, as the sampling component is so large in our case, leading to similar results of the two methods, we chose the simpler weighting matrix based on the community size only.

^5^We do not present detailed arguments on the shape of the relationship between mortality (the mortality gap) and the included variables. There exist a voluminous theoretical and empirical literature on these interactions on the individual and aggregated level. For a comprehensive review see [45].

^6^As the Austrian education system differs quite strongly from other countries, we also included in this "highest" level of education the degrees for primary and secondary school teachers and similar educations which formally do not belong to university degrees in Austria, but would yield a bachelor's degree according to international standards.

^7^We also ran regressions of the absolute change in mortality on initial (absolute) mortality to consider the fact that absolute improvements in health are probably more important from a health policy perspective than percentage changes in the health status. All our conclusions are unaffected in this specification, where the beta coefficients in all four cases are slightly higher (more negative) and the *R*^2 ^is higher than in our reported specification (varying between 0.58 and 0.67 for absolute beta-convergence).

^8^As the statistic is distributed as chi-squared (χ^2 ^= 2(*N *- 1)*RC*, where *N *is the number of communities and RC is the calculated Kendall rank concordance measure with *N *- 1 degrees of freedom) and we test the null hypothesis of no association between ranks of different years, the null can easily be rejected in all four cases.
